# Supplementary material for: SARS-CoV-2 nucleocapsid protein undergoes liquid-liquid phase separation stimulated by RNA and partitions into phases of human ribonucleoproteins
Source: bioRxiv. 2020 Jun 10:2020.06.09.141101. Preprint. [Version 1] doi: 10.1101/2020.06.09.141101 (PMC7302208; doi:10.1101/2020.06.09.141101)
Supplement: 1 [file NIHPP2020.06.09.141101-supplement-1.pdf]

bioRxiv preprint doi: https://doi.org/10.1101/2020.06.09.141101. this version posted June 10, 2020. The copyright holder for this preprint (which was not certified by peer review) is the author/funder. It is made available under a CC-BY 4.0 International license.

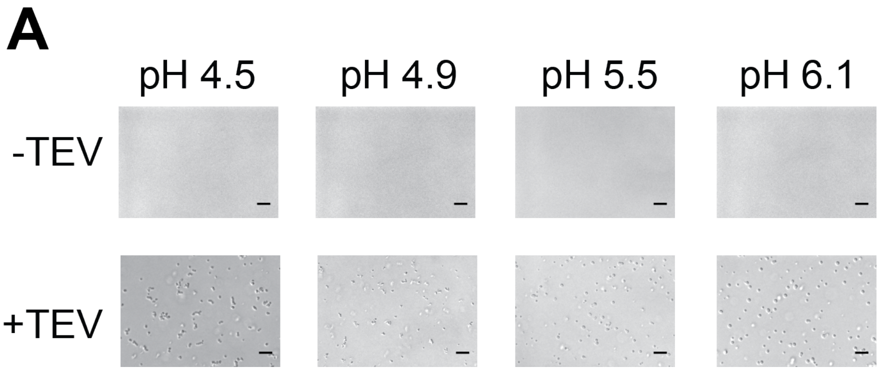

SI Figure 1: Low pH conditions induce aggregation of MBP-NP. A) DIC micrographs of 50 μM MBP-N in varying pH conditions. At lower pH conditions, droplets appear to be non-spherical, consistent with less fluid behavior. Scale bars represent 50 μm.

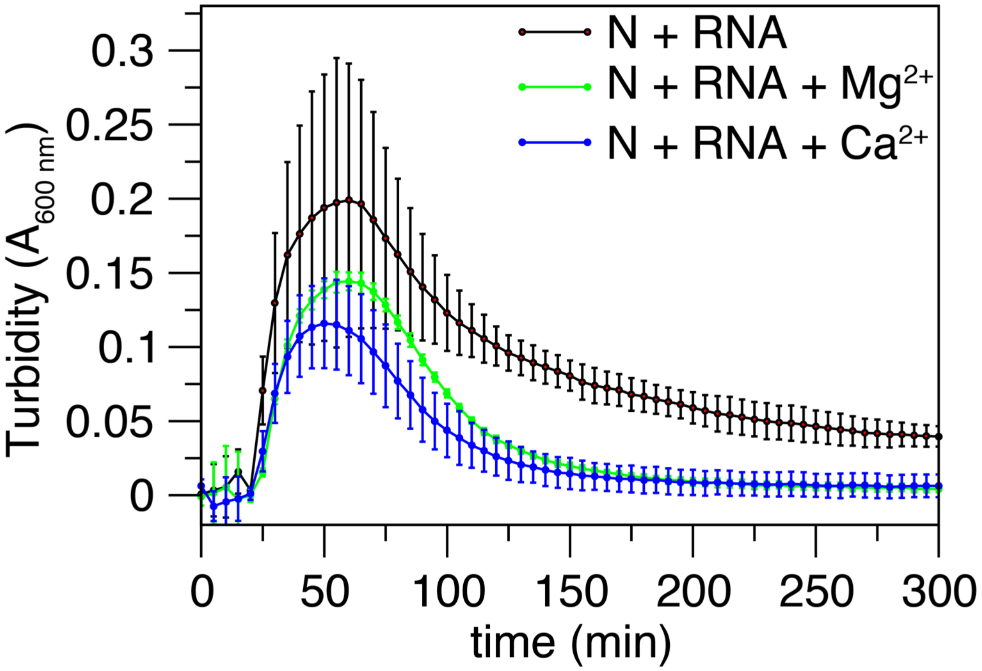

SI Figure 2: Divalent metal salts do not substantially alter N LLPS. Addition of 2 mM $MgCl_2$ or $CaCl_2$ does not alter LLPS of 50 μM MBP-N in the presence of 0.5 mg/mL RNA and 70 mM NaCl.
